# Passing Events and Topics

**Published:** 1921-02-12

**Authors:** 


### Passing Events and Topics.

### A Successful Hospital Ball.

The Royal Salop Infirmary Ball, which took place at Shrewsbury, realised over £1,100 for the infirmary.

### Domestic Science and Welfare Centres.

The County Welfare Officer for Durham desires to arrange a course of instruction in domestic science for mothers attending the Centres. There are over a hundred teachers of domestic science on the staff of the Education Committee, and she suggests the utilisation of the services of some of them at certain Centres.

### Gift from Ex-Service Men.

Under the United Services Fund the Llanelly and Burry Port branches of the Welsh Legion of Ex-Service Men received a sum of £1,283, and this they have handed over towards the Llanelly hospital extension. The ex-Service men, it should be added, have two representatives on the managing committee of the institution, a circumstance which greatly stimulates their interest in it.

### Hospital Saturday Fund Annual Dinner.

His Royal Highness the Duke of York has kindly consented to preside at the annual dinner of the Hospital Saturday Fund, at the Holborn Restaurant, on Saturday, February 12. Tickets are 10s. 6d. each, and the seating accommodation is strictly limited to 250. We are requested to state that evening dress is optional.

### A Flourishing Aid Society.

It is gratifying to learn from the report of the Leytonstone Branch of the Good Intent Hospital Aid Society that, in spite of the present difficult times, their work of the past year shows good progress. The total amount received was some £951, as against £534 in 1919; the number of letters issued has increased from 278 to 466; while the membership of the branch shows a steady growth.

### Extra Help or Overtime?

Faced with the alternative either of paying overtime to their nurses or of employing a larger staff, the Leyton District Council have adopted the report of their hospital Sub-Committee in favour of the latter plan, and propose to erect additional accommodation which is thus necessitated in order to secure the use of all available wards in their institution.

### Ambulances for Lying-in Patients.

The managers of the Mothers' and Infants' Welfare Centre in Argyll Square have called the attention of the St. Pancras Borough Council to the special difficulty of mothers in Kentish Town in finding conveyances to take them to the lying-in hospitals at night. The usual price of an ambulance is 10s., and is prohibitive to most of the mothers, and taxicabs are not procurable in the district. The Council has been asked if it could make some arrangements for the provision of conveyances free of charge, or at a small fee. The Council's Health Committee, however, think the subject is really one for the County Council, and suggests that that authority should be urged to provide the required service generally for London.

### Forthcoming Tuberculosis Conference.

The Conference of the International Union against Tuberculosis (Paris), arranged by the National Association for the Prevention of Tuberculosis, 20 Hanover Square, London, W. 1, will be held in London from July 26 to 28. Delegates from about twenty-five different countries will attend and take part in the proceedings. Professor Calmette will open a discussion on "The Modes of Diffusion of Tuberculosis throughout the Races of the World," and there will be three public addresses, given by a Frenchman, an American, and an Englishman. Visits to tuberculosis hospitals, sanatoria, laboratories, etc., will be arranged.

### Elswick Hospital.

Lancashire County Council has been negotiating for the purchase of the Elswick Sanatorium, but now the Hospital Board refuses to accept the offer submitted by the County Council. Now steps are being taken to secure the extra accommodation required by the County Council by providing for twenty-six additional beds at the High Carley Sanatorium and thirty-four additional beds at the Rufford Sanatorium.

### A Substantial Subscription.

Mr. Walter Morrison has paid his annual subscription of £5,000 to King Edward's Hospital Fund for London.

